# Bullous Pemphigoid Associated With the Use of Sacubitril/Valsartan: A Case Report and Literature Review

**DOI:** 10.7759/cureus.53160

**Published:** 2024-01-29

**Authors:** Daniel A Martin Arsanios, Lina M Gómez-Álvarez, Natalia Muñoz-Angulo, Claudia Montealegre, Elias Quintero Muñoz, Carlos Calderón-Vargas

**Affiliations:** 1 Internal Medicine, Hospital Universitario de La Samaritana, Bogotá, COL; 2 Internal Medicine, Universidad de La Sabana, Bogotá, COL; 3 Internal Medicine, Universidad El Bosque, Bogotá, COL; 4 Internal Medicine, Fundación Universitaria Juan N. Corpas, Bogotá, COL

**Keywords:** adverse reactions, skin lesions, blisters, sacubitril/valsartan, bullous pemphigoid

## Abstract

Bullous pemphigoid (BP) is a complex autoimmune blistering disease with an increased incidence in the comorbid population, particularly among older adults. The occurrence of drug-induced BP is associated with an underlying genetic predisposition, triggering an enhanced immune response, the formation of autoantibodies, and alterations in antigenic properties within the basement membrane zone. With over 90 identified drugs capable of precipitating BP, we present the case of an 87-year-old woman with comorbidities who experienced a medication change from losartan to sacubitril/valsartan. Three months later, erythematous lesions appeared on her lower limbs, progressing to a generalized rash accompanied by itching. Over the following month, these lesions evolved into tense blisters with serous content and intense pain. Suspecting the medication switch to sacubitril/valsartan as the cause, the drug was discontinued, and immunomodulatory treatment was initiated, resulting in a notable improvement in the lesions.

## Introduction

Bullous pemphigoid (BP) is a complex autoimmune blistering disorder, primarily affecting older adults [1,2]. The main clinical manifestations involve the development of tense vesiculobullous lesions on an erythematous, annular, urticarial, or eczematous base, accompanied by crusts and erosions. Notably, BP does not exhibit a gender predilection, with an incidence ranging from 0.2 to 3 new cases per 100,000 inhabitants [2,3]. In the United States and Central Europe, the annual incidence can reach up to 13 new cases per million people [1,3,4].

Drug-induced BP is characterized by the generation of B cell clones recognizing autoantigens, leading to antibody production. Medications containing thiol groups, such as captopril, penicillins, furosemide, cephalosporins, acetylsalicylic acid, and valsartan, are frequently associated with the development of this pathology [1-4].

The onset of drug-induced BP is linked to an inherent genetic predisposition, resulting in heightened immune responses, autoantibody generation, and changes in antigenic properties against the basement membrane zone (BMZ) [3-5]. This phenomenon occurs through interactions between drug antibodies and active principles or excipients [1,4,5]. Diagnosing drug-induced BP can be challenging, especially in patients with polypharmacy. However, it has been observed that, in general, lesions appear approximately two to three months after exposure to the drug, presenting as a highly pruritic and widespread bullous eruption without mucosal involvement [2,3,5].

Over 90 drugs capable of triggering a response against the BMZ have been identified, underscoring the importance of describing key clinical manifestations and exploring potential pathophysiological processes in previously unreported cases of drug-induced BP [1,4,5]. In this context, we present the first documented case in the literature of BP secondary to the use of sacubitril/valsartan.

## Case presentation

An 87-year-old woman, diagnosed with ischemic heart disease, heart failure featuring reduced ejection fraction, hypertension, and atrial fibrillation, had been receiving a medication regimen comprising losartan, carvedilol, atorvastatin, dapagliflozin, and rivaroxaban for two years. However, three months before her hospitalization, losartan was discontinued, and sacubitril/valsartan was initiated. Upon presenting to the emergency department, she reported a three-month history of clinical symptoms that commenced with erythematous lesions on her lower limbs, progressively spreading in a generalized manner. These lesions induced itching and evolved over a month into tense blisters filled with serous content, accompanied by intense pain. Despite outpatient management involving crotamiton, amoxicillin, and silver sulfadiazine, there was no observed improvement.

**Figure 1:**
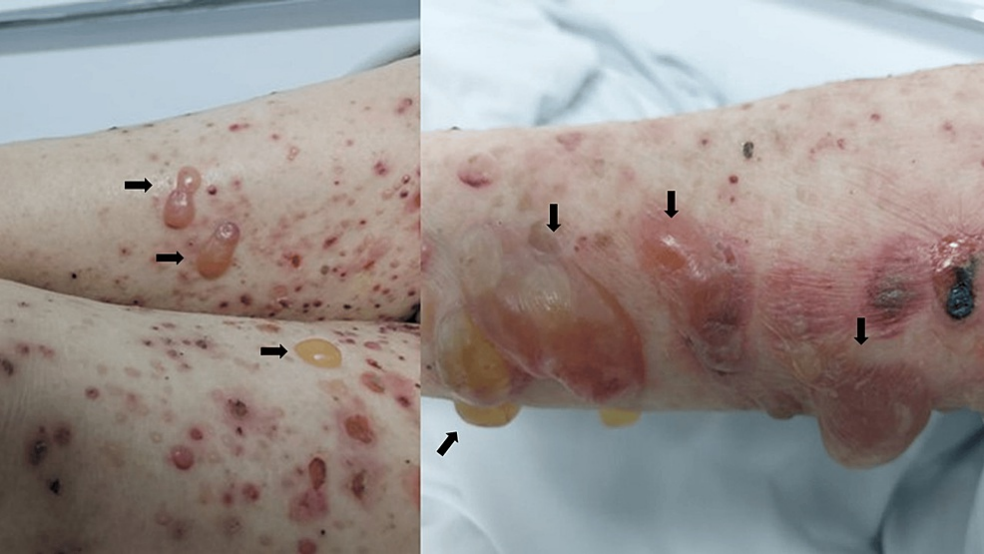
General characteristics of BP

**Figure 2:**
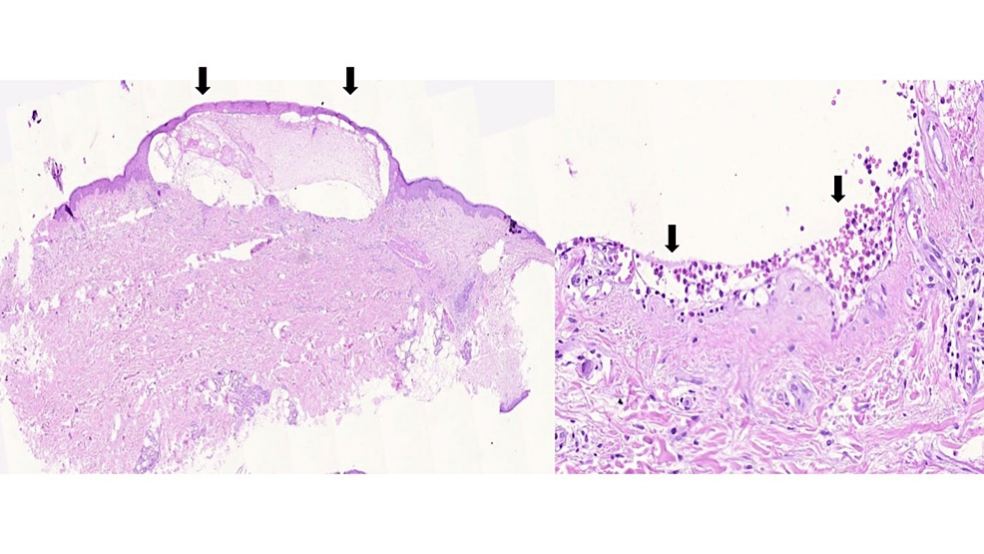
Skin biopsy Detachment at the dermoepidermal junction with fibrin and eosinophils, mild edema in the remaining dermis, and perivascular lymphohistiocytic infiltration with eosinophils. Immunofluorescence was positive for IgG and C3 in a punctate pattern at the basement membrane, confirming the diagnosis.

Upon admission, the patient exhibited vital signs within the normal range. The physical examination unveiled numerous erythematous-violaceous, edematous plaques characterized by tense blisters ranging from 1 to 4 cm in size. Predominantly affecting the upper limbs, torso, and thighs, these lesions were covered by serous and bloody crusts. Furthermore, multiple excoriations were noted on the eyelids, retroauricular region, and abdomen, which account for 55% of the total body surface area (refer to Figure 1). Biopsies taken from the lesions revealed detachment at the dermoepidermal junction with the presence of fibrin and eosinophils (refer to Figure 2). Notably, hematological, hepatic, and renal function tests yielded results within normal parameters at admission. Comprehensive screening tests for human immunodeficiency virus, hepatitis B and C viruses, syphilis, tumor markers, cytology, mammography, and contrast-enhanced CT scans were conducted to identify potential secondary causes, and all reported results were within the normal range.

The diagnostic suspicion of BP induced by Sacubitril/Valsartan prompted the discontinuation of the medication. Subsequently, pulses of corticosteroids with methylprednisolone at a dosage of 1 g per day for three days were initiated. Following this, the patient was maintained on prednisolone at a dose of 1 mg/kg/day, azathioprine at 2 mg/kg/day, and antihistamines, resulting in a significant improvement in the lesions. Molecular tests or genetic profiles were not conducted to definitively rule out a potential association with gene mutations.

## Discussion

This manuscript delineates the distinctive features of a patient with BP induced by sacubitril/valsartan. Multiple erythematous-violaceous and edematous plaques with tense blisters were evident. Notably, the discontinuation of sacubitril/valsartan therapy and initiation of a therapeutic regimen, including corticosteroids and antihistamines, resulted in a marked improvement in cutaneous lesions. A biopsy revealed detachment at the dermoepidermal junction with a significant eosinophilic content, a characteristic feature associated with the BP triggered by medications. Identification of the recently initiated drug established a causal relationship with BP, prompting the discontinuation of the triggering medication. It is highly likely that this adverse effect is linked to valsartan, based on previous case reports associating this pharmacological group with BP development. However, the possibility that both the combination and sacubitril are involved in the onset of these cutaneous lesions cannot be completely ruled out. To date, no specific cases of BP related exclusively to the use of sacubitril/valsartan have been reported [4-6].

The mechanism of action of sacubitril/valsartan involves the concurrent inhibition of neprilysin through its active metabolite, sacubitril (LBQ657), and antagonism of the angiotensin II subtype AT-1 receptor by valsartan [7-9]. The precise mechanism by which this drug induces BP is not fully understood. Moreover, it is postulated that this induction could be related to the drug's ability to modulate or enhance the immune response or cause alterations in antigens at the dermoepidermal junction in individuals with specific genetic predispositions [1,4,7,8]. This process initiates with the drug binding to molecules present in the lamina lucida of the basement membrane, triggering an antigenic action associated with BPAG1, BP230, or BP180 antigens, with the latter being most linked to disease development. The involvement of antibodies activated by polymorphonuclear cells, generating proteolytic enzymes, has also been observed. Furthermore, the presence of anti-BP180NC16A or anti-BP230 autoantibodies has been reported in patients who developed the disease, compared to those who did not receive drugs, supporting the theory of epitope spreading [4,6-8].

This is typically an exclusion diagnosis related to systemic or topical drug use, with a clinical, histological, and immunopathological presentation similar to the idiopathic form [1,2,6]. It is characterized by urticarial and eczematous lesions, highly pruritic, followed by the later appearance of large tense blisters containing serous or hemorrhagic fluid, predominantly on the lower trunk, extensive folds, and flexor surfaces of limbs, tending to spare the cephalic pole, and mucosal involvement is very infrequent and mild when present [2,6]. The histological diagnosis with the presence of eosinophils aligned at the basement membrane, and the presence of IgG and C3 deposits in it are indicative of this entity [1,4,6]. Similarly, the disease course was self-limiting, and the discontinuation of the medication along with corticosteroid therapy resolved all the described lesions. One of the main limitations in the diagnosis of the clinical case lies in the absence of genetic mutation tests. Likewise, the lack of long-term clinical follow-up prevents the comparison of clinical signs and the demonstration of their complete resolution.

Drug-induced BP is an exclusion diagnosis arising in relation to systemic or topical administration of a drug, presenting clinical, histological, and immunopathological manifestations similar to the idiopathic form [1,2,4]. It is characterized by the presence of highly pruritic urticarial and eczematous lesions, followed by the appearance of large tense blisters containing serum or blood, mainly on the lower trunk, extensive folds, and flexor surfaces of the limbs. Usually, it spares the cephalic pole, and mucosal involvement is infrequent, being mild if present [1,4,6]. In the present case, there were no findings in the physical examination or temporal elements that would differentiate it from classical BP. The histological diagnosis, with the presence of eosinophils aligned at the basement membrane and deposits of immunoglobulin G and complement 3 in it, remains indicative of this entity [2,5,6]. Additionally, the disease course was self-limiting, and discontinuation of the medication, combined with corticosteroid administration, led to a complete resolution of the described lesions.

## Conclusions

A patient exhibiting BP induced by sacubitril/valsartan was observed, displaying numerous erythematous-violaceous and edematous plaques accompanied by tense blisters. The biopsy results indicated detachment at the dermoepidermal junction with a notable eosinophilic presence. Ultimately, discontinuation of the therapy, coupled with the initiation of a treatment plan incorporating corticosteroids and antihistamines, resulted in a significant amelioration of cutaneous lesions.
